# Relationship between Photosynthetic Capacity and Microcystin Production in Toxic *Microcystis Aeruginosa* under Different Iron Regimes

**DOI:** 10.3390/ijerph15091954

**Published:** 2018-09-07

**Authors:** Xun Wang, Peifang Wang, Chao Wang, Jin Qian, Tao Feng, Yangyang Yang

**Affiliations:** 1 Key Laboratory of Integrated Regulation and Resource Development on Shallow Lakes, Ministry of Education, Hohai University, Nanjing 210098, Jiangsu, China; xwang2014@hhu.edu.cn (X.W.); cwang@hhu.edu.cn (C.W.); hhuqj@hhu.edu.cn (J.Q.); tfeng@hhu.edu.cn (T.F.); yangyy@hhu.edu.cn (Y.Y.); 2 College of Environment, Hohai University, Nanjing 210098, Jiangsu, China

**Keywords:** cyanobacterial growth, iron, microcystin production, *Microcystis aeruginosa*, photosynthetic capacity

## Abstract

Blooms of harmful cyanobacteria have been observed in various water bodies across the world and some of them can produce intracellular toxins, such as microcystins (MCs), which negatively impact aquatic organisms and human health. Iron participates significantly in cyanobacterial photosynthesis and is proposed to be linked to MC production. Here, the cyanobacteria *Microcystis aeruginosa* was cultivated under different iron regimes to investigate the relationship between photosynthetic capacity and MC production. The results showed that iron addition increased cell density, cellular protein concentration and the Chl-a (chlorophyll-a) content. Similarly, it can also up–regulate photosynthetic capacity and promote MC–leucine–arginine (MC–LR) production, but not in a dose–dependent manner. Moreover, a significant positive correlation between photosynthetic capacity and MC production was observed, and electron transport parameters were the most important parameters contributing to the variation of intracellular MC–LR concentration revealed by Generalized Additive Model analysis. As the electron transport chain was affected by iron variation, adenosine triphosphate production was inhibited, leading to the alteration of MC synthetase gene expression. Therefore, it is demonstrated that MC production greatly relies on redox status and energy metabolism of photosynthesis in *M. aeruginosa*. In consequence, more attention should be paid to the involvement of photosynthesis in the regulation of MC production by iron variation in the future.

## 1. Introduction

In recent decades, the frequent outbreak of cyanobacterial blooms in aquatic ecosystems has become a public and ecological concern worldwide. Many environmental factors including nutrient concentrations and climatic variables are reported to influence cyanobacteria growth [1,2,3], and the adaptation of cyanobacteria to environmental variation is also an important factor affecting cyanobacteria proliferation [4,5]. As a dominant cyanobacterial species, *M. aeruginosa* (*Microcystis aeruginosa*) is able to produce secondary metabolites, such as hepatotoxin microcystins (MCs) which can lead to liver necrosis in acute doses and hepatocellular carcinoma in chronic low doses [6]. Over 240 MC variants were reported so far [7,8] and microcystin–leucine–arginine (MC–LR) is among the most common variants [9] with a drinking water guideline (≤1 μg L^−1^) set by the World Health Organization [10]. Recent research has focused on MC environmental behaviors and MC accumulation and toxicity in various organisms [11,12], while the effects of environmental factors on MC production remain unidentified [13,14,15].

According to previous studies, MC was proposed to act as an iron–scavenging molecule [16,17]. Growth comparisons between toxin–producing and non–toxin–producing strains of *M. aeruginosa* suggested that MC may contribute to the more efficient uptake or storage of iron [18] as MC producers can remain feasible for longer time during iron limitation [19]. It has also been suggested that MC is chelated with iron inside algal cells and is responsible for the inactivation of free cellular iron [20]. However, different propositions have also reported that MC protects toxin–producing strains from iron stress and subsequent reactive oxygen species–induced damage [17]. This is supported by the study of Zilliges et al. [21], who observed increased binding between MC and redox-related proteins under iron variation. However, the mechanism between iron and MC production still remains unclear [22]. Iron is one of the essential micronutrients for algae due to its important role in many metabolic functions, such as chlorophyll-*a* (Chl-*a*) synthesis, photosynthetic electron transport, respiration and nutrient uptake [23,24]. According to the World Health Organization, the iron concentration ranges from 0.5 to 50 mg L^−1^ in natural waters [25]. Although dissolved total iron concentrations may be high in waters, its bioavailability does not act the same, and many lakes are considered iron–deficient, including Lake Erie in North America [26], Lake Erken in Sweden [27] and Lake Taihu in China [28,29,30], which might be restrictive to algal growth and impact photosynthetic pathways [31]. In order to investigate the iron threshold of algal growth, many researchers have conducted batch experiments in the laboratory. Alexova et al. [17] reported that MC production in *M. aeruginosa* was limited under low iron concentration (10, 100 and 1000 nM, EDTA–complexed iron) and Li et al. [32] observed the stimulation of iron on growth and MC production of cyanobacteria at the highest iron concentration (5 μM, FeCl_3_). As the fourth most abundant element, iron accounts for 5% of the earth’s crust [33], and naturally, it may enter into water during rain wash of soils and sediments [34]. The distributed forms can also influence iron occurrence since it usually exists as a dissolved ion compound, a particulate compound or an organic coordination complex in waters [34]. However, recently, excess iron was introduced into water through anthropogenic wastewater discharge, especially from effluents of iron and steel industries [35], increasing the potential of iron–replete occurrence in freshwaters. Nevertheless, the majority of studies focused on the response of MC production under iron–limited conditions while few studies have been conducted in iron-replete setup [36].

Cyanobacteria are recognized as autotroph species which can transform light into chemical energy through photosynthesis [37]. The photosynthetic components, including the photosystem II (PS II) reaction center, contribute significantly to photosynthesis of cyanobacteria [38]. As revealed by many studies, iron is closely related to the photosynthetic process in cyanobacteria in natural environments [36,39]. However, being the two vital processes affected by iron variation, the link between photosynthesis and MC production was not clearly confirmed, although some studies have proposed the possibility. According to the statistical study of Jiang et al., iron and photosynthesis were reported to have a significant interactive effect on MC production [40] and the involvement of photosynthesis in MC production was also observed by the evaluation of static linear and dynamic non-linear models [41]. Moreover, it is reported that MCs can bind to intracellular photosynthesis-related proteins as a toxin-storage strategy [21,42]. Immunogold–labelling results showed that a large proportion of intracellular MC was combined with the thylakoid region where PS II [21,43] is located, which supports a possible link between MC and photosynthesis. Furthermore, a link between MC production and photosynthesis was also suggested as the regulation of MC genes and MC production by light appeared to be universal among cyanobacteria [44]. Fortunately, in recent decades, pulse amplitude modulated fluorometry (PAM) has contributed to photosynthetic apparatus assessment [45], which may facilitate the determination of a relationship between photosynthesis capacity and MC production.

In the present study, the toxic strain *M. aeruginosa* was cultivated under various iron regimes (control, iron–limited and iron–replete). Cyanobacterial growth and MC production, as well as the altered expression of *F_v_*/*F_m_*, *rETR_max_* and α, were investigated. Given the potential occurrence of iron variation in freshwaters, our study aims to reveal iron effects on MC production and complete our knowledge about MC biosynthesis regulation, which could improve water management strategies to reduce cyanobacteria–derived water quality issues.

## 2. Materials and Methods

### 2.1. Cyanobacteria Cultivation and Experimental Setup

The *M. aeruginosa* strain was provided by the Freshwater Algae Culture Collection of the Institute of Hydrobiology in Wuhan, China (FACHB–905), and pre–cultivated in standard BG–11 medium. During our experiment, *M. aeruginosa* was cultivated in a modified BG–11 medium with the initial pH of 8.0 [46]. In the modified BG–11 medium, the concentration of ammonium ferric citrate was set to 0 (control), 10, 20, 40, 60, 80 and 100 μM. Calculation by MINEQL+, a program for equilibration of chemical species in solution, verified that pFe (–lg[free ferric]) corresponded to the total iron concentration in each treatment, and precipitation of other trace metals was negligible under the conditions employed. The experimental period lasted for 10 days, which include the initial lag stage and the logarithmic stage of *M. aeruginosa* growth according to our previous experiment [46]. In order to simulate the iron effects on phytoplankton under both iron-limited and iron–replete conditions, seven concentration gradients were selected to represent the control (0 μM), iron–limited (10, 20 μM) and iron–replete (40, 60, 80 and 100 μM) conditions. Both citrate and EDTA were used as chelators to keep iron in solution during the experiment [24]. Each iron exposure was performed in triplicate with an initial cell density of 2 × 10^5^ cells mL^−1^. All measures were also conducted in triplicate.

The cultivation was conducted in Erlenmeyer flasks (1 L) at 25 °C [47] in batch mode with a photosynthetically active radiation of 30 μmol photons m^−2^ s^−1^ under a 12 h /12 h regime. The experimental apparatus and culture medium were sterilized by autoclaving (121 °C, 30 min, MLS–3750, Sanyo, Japan) to prevent bacterial contamination. All Erlenmeyer flasks were shaken manually every day to avoid cell aggregation and were reorganized randomly to reduce irradiance differences.

### 2.2. Determination of Cyanobacterial Growth and Photosynthetic Capacity

The cell number of *M. aeruginosa* was counted daily under a light microscope (Axioskop 40, Carl Zeiss, Germany). The chlorophyll-*a* (Chl-*a*) content was measured using a phytoplankton analyzer (Phyto–PAM, Hein Walz GmbH, Effeltrich, Germany) by pouring a 3 mL sample solution into a measuring chamber after dark adaptation of 10 min. Then, after the stabilization of 30 s under modulated (non–actinic) light, the Chl–*a* content was read in Phyto–Win Software (version 1.45, Hein Walz GmbH, Effeltrich, Germany).

The protein content of *M. aeruginosa* cells was measured daily using the Bradford method [48]. The cells were harvested from 10 mL cyanobacterial solution by low vacuum filtration using a 0.22 μm Whatman GF/C glass microfiber filter and were re–suspended in 20 mL phosphate buffered saline solution (PBS, 0.05 M, pH = 7.8). Then, they were disrupted by an ultrasonic cell pulverizer (1200-98, BioSafer, Nanjing, China) for 15 min and were surrounded by ice bags to avoid overheating. After centrifugation (10,615× *g*, 4 °C, 10 min, H2050R–1, Xiangyi, Hunan, China), the supernatant was used for protein content measurement.

Similar to Chl–*a*, *F_v_*/*F_m_*, *rETR_max_* and *α* can also be measured in vivo by the Phyto–PAM analyzer according to the method of Garrido et al. [49]. Prior to measurement, a 10 min dark adaptation period was also required. The three photosynthetic capacity parameters can be calculated as:

(1) $$F_{v}/F_{m}=\left(F_{m}-F_{0}\right)/F_{m}$$

(2) rETR=ϕPSII×PAR×0.84×0.5

(3) $$\alpha =rETR_{max}/E_{k}$$

where *F_m_* is the maximum fluorescence yield after dark acclimation, *F*_0_ is the minimal fluorescence yield after dark acclimation, *F_v_* is the variable fluorescence, *rETR* represents the relative electron transport rate, *ϕPSII* is the effective quantum yield in PS II, *PAR* is the photosynthetically active radiation and *E_k_* is the minimum saturating irradiance. The units were μmol electrons m^−2^ s^−1^/μmol photons m^−2^ s^−1^ for *α* and μmol electrons m^−2^ s^−1^ for *rETR_max_,* respectively.

### 2.3. Extraction and Measurement of MC–LR

Cells from a 30 mL cyanobacterial solution were separated by low vacuum filtration using 0.22 μm Whatman GF/C glass microfiber filter, and the filtrate was used for the subsequent analysis of extracellular MC–LR content. The collected cells in the filter were rinsed and re–suspended in deionized water prior to the 15 min ultrasonic disruption (1200–98, Biosafer, Nanjing, China). The disrupted algal solution was centrifuged (8600× *g*, 10 min, 4 °C), and the supernatant was concentrated by solid–phase extraction (SPE) cartridges which were pre–activated by methanol (CNWBOND LC–C18, ANPEL, Shanghai, China). The MC–LR in the cartridges, presenting the intracellular toxin, was eluted by methanol. The eluent was dried in nitrogen prior to the adjustment of volume to 1 mL using HPLC–grade methanol.

The details about MC–LR measurement can be found in our previous study [46]. Briefly, the MC–LR concentration was measured by a HPLC system (Waters Alliance e2695 and Waters 2489 UV/Visible detector, Milford, MA, USA) and the wavelength was set to 238 nm. The injection volume was 50 μL with a 0.6 mL min^−1^ flow rate. The separation was carried out on an XBridge LC C18 HPLC column (XBridge Systems, Inc., Mountain View, CA, USA). The mobile phase was constituted by deionized water (solvent A, 40%) and HPLC–grade methanol (solvent B, 60%, Tedia, Fairfield, OH, USA), and they both contained 0.1% formic acid (Merck, Germany). The standard MC–LR (Agent Technology, Lausen, Switzerland) was stored at −25 °C and the standard curve of MC–LR (R^2^ > 0.999) was established ranging from 0.5 to 1000 μg L^−1^.

### 2.4. Generalized Additive Model (GAM) Construction and Statistical Analysis

GAM is a non–parametric extension of a Generalized Linear Model (GLM) and it uses a link function to establish the relationship between the mean of the response variable and a smoothed function of the predicted variables [50]. Therefore, GAM is flexible in model structure and can deal with non–linear and non–monotonic response curves between photosynthetic capacity parameters and intracellular MC–LR concentration compared with other statistical models [51]. The general form of GAM is:

(4) $$g\left(E\left(Y\right)\right)=\beta_{0}+f_{1}\left(X_{1}\right)+f_{m}\left(X_{m}\right)$$

where *E*(*Y*) is the expectation of the response variable *Y*, *g*(.) is the link function, *β*_0_ is the intercept and *f_j_*(*.*) is the smoothing function of the predictor variables *X_i_*. In our study, the GAM was set up by using the measured photosynthetic data to quantify the non–linear relationship between photosynthetic capacity parameters and intracellular MC–LR concentration. Details concerning the steps of construction were described in previous studies [51,52]. Briefly, the building of GAM consisted of two steps: (1) Preparative analysis of the relationship between photosynthetic capacity parameters and MC production, which identified the distribution type of intracellular MC–LR data. (2) Setup and choice of model: constructed several equivalent models and selected an appropriate model by analysis of variance. The steps of GAM construction were all calculated in R (version 3.2.1, Vienna, Austria) with the package mgcv.

The average value of three replicates and the standard deviation (SD) were calculated in our study. One–way ANOVA followed by Tukey’s test was applied to identify the significant differences (*p* < 0.05). The statistical analyses were carried out with SPSS (version 17.0, Chicago, IL, USA) and packages vegan and mgcv of R (version 3.2.1, Vienna, Austria).

## 3. Results and Discussion

### 3.1. Response of Growth and Photosynthetic Capacity of M. aeruginosa to Iron Variation

**Figure 1:**
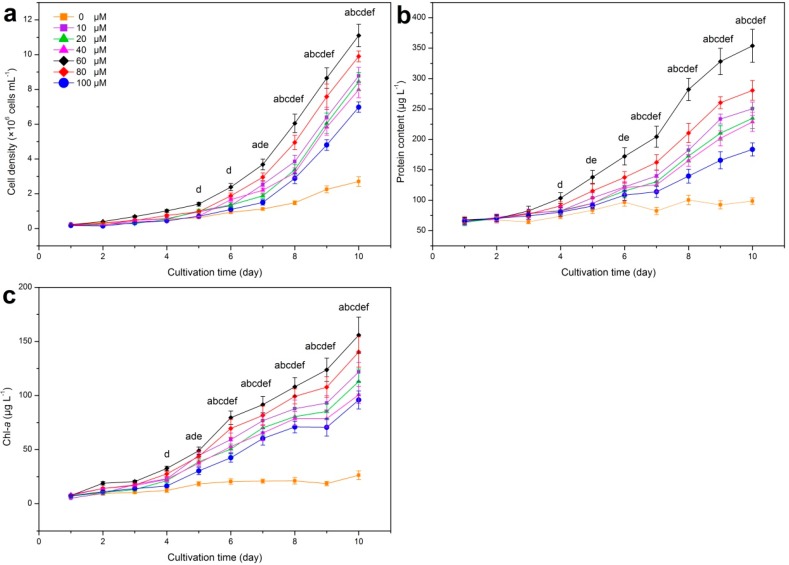
Cell density (**a**), protein content (**b**) and Chl-*a* (chlorophyll-*a*) content (**c**) of *M. aeruginosa* (*Microcystis aeruginosa*) FACHB–905 under different iron conditions. Error bars represent the standard deviation of triplicate samples. a, significant difference between 10 μM exposure and control exposure on the same cultivation day; b, 20 μM–0 μM; c, 40 μM–0 μM; d, 60 μM–0 μM; e, 80 μM–0 μM; f, 100 μM–0 μM. *p* < 0.05 was accepted as statistically significant for differences.

The growth of *M. aeruginosa* under different iron conditions is presented in Figure 1. It was observed that the cell density, protein content and Chl–*a* content in all treatments were invariant at the very early stage of cultivation. According to previous studies, it will take about 4 days for *M. aeruginosa* to consume the pre–stored iron in cells under iron–deficient conditions [18,53], which was also observed in our study. Moreover, cell density of *M. aeruginosa* under iron–containing exposure increased significantly compared with that in the control exposure (*p* < 0.05), while the control exposure (0 μM) possessed the lowest cell density with the ultimate value of 3 × 10^6^ cells mL^−1^, implying that iron addition can promote cell density. In addition, cell density stayed in the highest position in the 60 μM exposure rather than in 100 μM exposure during the whole experiment, indicating that the stimulation by iron was not in a dose–dependent manner. Although the positive effect of iron on cyanobacterial growth has been confirmed, the promoting threshold of iron varied in different research. It is reported by Wang et al. that the growth rate of *M. aeruginosa* was inhibited under 12.3 μM and over 24.6 μM iron [28], while 100 μM iron was observed to stimulate *M. aeruginosa* cell growth in the study of Xing et al. [36]. As a result, standardized experimental conditions in laboratory are essential for the identification of iron threshold for algal growth, the results of which can contribute to cyanobacterial bloom controls under different iron conditions in field environments in the future. In addition to cell density, it is commonly recognized that protein participates in almost all physiological functions in cells, leading to its indicative role in cyanobacterial growth evaluation [54,55]. In order to investigate the effects of iron variation on protein synthesis of *M. aeruginosa*, the protein content during the experiment was measured and is presented in Figure 1b. Similar to cell density, compared with the control exposure, the protein content under iron–containing exposure was significantly stimulated by iron addition and the promotion was not in a dose–dependent manner.

Chl-*a* is one of the main photosynthetic pigments that can indicate the primary productivity of aquatic systems [56], contribute to the green color of *M. aeruginosa* cells, assist light–harvesting process and transfer energy in photosystems [57,58]. Consequently, it presents both the current cyanobacterial biomass and the potential productivity of *M. aeruginosa* [30,59,60]. It was observed in our study that rapid chlorosis of cells occurred in the control exposure at the early stage of cultivation, and the Chl-*a* content stayed invariant during the whole cultivation in this exposure (Figure 1c), suggesting that the iron–depleted condition inhibited Chl-*a* biosynhesis and damaged the photosynthesis process in *M. aeruginosa*. According to previous studies, iron can control chlorophyll synthesis by affecting the formation of protochlorophyllide from Mg–protoporphyrin, which is catalyzed by the Fe–containing enzyme coproporphyrinogen oxidase [61], and chlorophyll is required by photosynthetic organisms for its light trapping and energy transduction activities [62]. Thus, it is not surprising that iron–starved exposure resulted in degraded photosynthetic machinery of cyanobacteria in our study, which was also reported by Alexova et al. [19]. Similar to cell density and protein content, the highest Chl-*a* content on the 10th day occurred at 60 μM exposure, peaking at 155.87 μg L^−1^, rather than under 100 μM exposure. According to previous studies, the dose–dependent relationship between iron addition and algal growth was often observed in many cyanobacteria, such as *Microcystis viridis* and *Microcystis wesenbergii* [36,63]. However, in our study, the non–dose–dependent promoting effect of iron addition on *M. aeruginosa* growth was observed.

**Figure 2:**
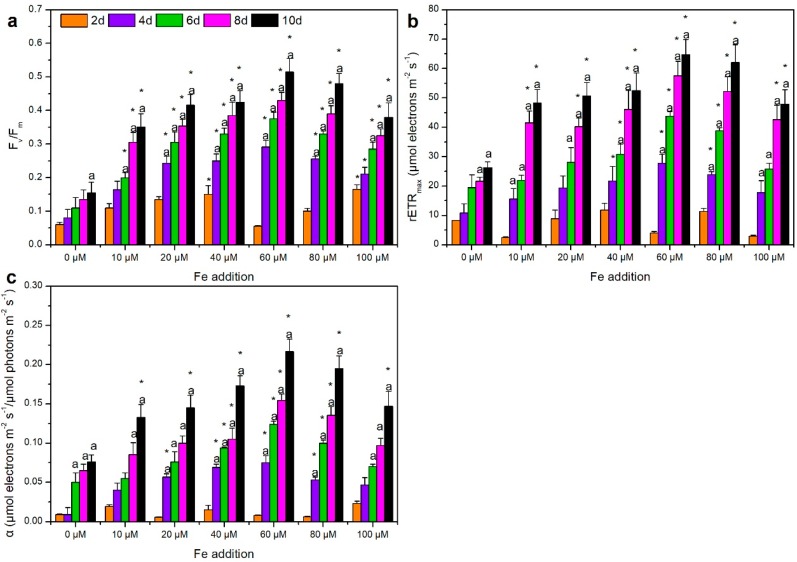
Photosynthetic parameters of *M. aeruginosa* (*Microcystis aeruginosa*) exposed to different iron concentrations ((**a**): *F_v_*/*F_m_*, maximal quantum yield, dimensionless; (**b**): *rETR_max_*, maximal relative electron transport rate, μmol electrons m^−2^ s^−1^; (**c**): *α*, photosynthetic efficiency, μmol electrons m^−2^ s^−1^/μmol photos m^−2^ s^−1^). Error bars represent the standard deviation of triplicate samples. a Significant difference between the sampling day and the 2nd day under the same iron exposure; * Significant difference between the iron addition exposure and the control exposure on the same cultivation day. *p* < 0.05 was accepted as statistically significant for differences between exposure data.

Revealed by previous studies, photosynthesis can convert the captured light energy into chemical energy and maintain physiological processes in cells [64,65]. In our study, the variation of photosynthetic parameters was presented in Figure 2. The maximal quantum yield *F_v_*/*F_m_* demonstrates the cell ability to convert light energy into chemical energy after the dark–adapted process [66]. The maximal relative electron transport rate *rETR_max_* is an approximation of the electron rate pumped in the photosynthetic chain, and the light capture efficiency, α, presents the maximum light–limited photosynthesis rate [67]. In our study, *F_v_*/*F_m_*, *rETR_max_* and α all increased significantly under iron–containing exposure (*p* < 0.05) (Figure 2). Since *F_v_*/*F_m_* and *rETR_max_* are both restricted by electron transport capacity or Calvin activity in photosynthesis [45], it is suggested that iron addition can stimulate photosynthetic capacity through the enhancement of electron transport chain, energy transfer, photon transfer and trapping capacity in photosynthetic process. Similar results have been observed in phytoplankton community, including cyanobacteria, i.e., that positive responses of photosynthetic parameters occurred following the supply of iron in Pacific Ocean [39]. Moreover, it is also reported that photosynthetic capacity is determined by the functional availability of photosynthetic components, which can be altered or damaged in response to iron variation [68,69], which was confirmed by, the chlorotic cells in the control exposure in our study. In addition, the three photosynthetic capacity parameters were all up–regulated in a dose–dependent manner under four exposures (10, 20, 40 and 60 μM) with the highest value of 0.52, 64.75 μmol electrons m^−2^ s^−1^ and 0.22 μmol electrons m^−2^ s^−1^/μmol photons m^−2^ s^−1^ under 60 μM exposure, respectively. Interestingly, as the highest stimulation of cell density, protein content and Chl-*a* content were all observed under 60 μM exposure, it can be speculated that the growth of *M. aeruginosa* cells is more active in this exposure. However, under 80 μM and 100 μM exposures, photosynthetic capacity was lower than that under 60 μM exposure and exhibited dose–suppression effect, demonstrating an impaired electron transport, leading to surplus electron accumulation [70] compared with lower exposures.

### 3.2. Iron Effect on MC Production in M. aeruginosa

**Figure 3:**
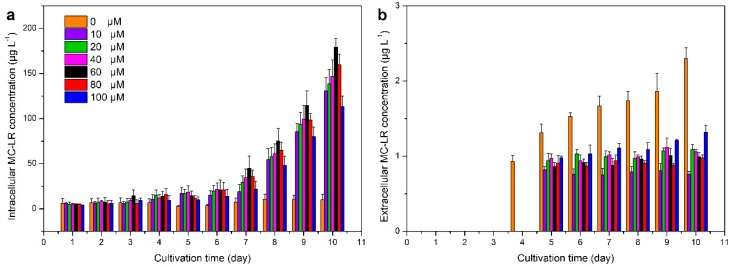
Intracellular (**a**) and extracellular (**b**) MC–LR concentration of *M. aeruginosa* (*Microcystis aeruginosa*) FACHB–905 under different iron conditions. Error bars represent the standard deviation of triplicate samples.

The intracellular MC–LR in iron–containing exposures increased markedly compared with that under control exposure with the peaks of 130.83, 138.76, 147.04, 179.14, 159.69 and 113.25 μg L^−1^ under 10, 20, 40, 60, 80 and 100 μM exposure, respectively (Figure 3a), indicating that iron addition can stimulate the production of MC–LR as observed in previous studies [17,46,71]. Moreover, the intracellular MC–LR concentration at all exposures showed similar variation in the first 4 days, while they behaved differently afterwards, suggesting that *M. aeruginosa* cells consumed stored iron and started to respond to extracellular iron stresses after 4 days [18,53]. Consistent with our result, Sinang et al. [29] also reported that total dissolved iron was strongly positively correlated with intracellular MC concentration. However, in our study, the 60 μM exposure exhibited the highest intracellular MC–LR concentration, implying that the hypothesized linear relationship between iron concentration and MC–LR production is not a straightforward one [72].

Because of the cyclic heptapeptide structure of MCs, they are too large to naturally pass through the cell membrane [7]. According to previous studies, although an adenosine triphosphate (ATP) binding cassette (ABC) transporter for MC transport, *mcy*H, was postulated to be related to MC production process [43], limited evidence of the active transportation of MC has been reported so far, indicating that the majority of MC–LR in the external environment was released after cell lysis [17]. In our study, the extracellular MC–LR was not detected until the 4th day (Figure 3b), demonstrating little occurrence of cell lysis in the early stage of cultivation. Moreover, different from intracellular MC–LR, extracellular MC–LR concentration fluctuated at a much smaller scale in all treatments (Figure 3b) during the experiment. The order of magnitudes also differed remarkably in intra– and extracellular MC–LR concentrations. Consequently, it is deduced that the majority of MC–LR was kept inside the cells during our experiment and little MC–LR was released into external environment. Several studies have also reported that maximum MC concentrations were not recorded until the end of the growth cycle or during bloom collapse [73]. Therefore, iron addition could actually increase the intracellular toxin production but may not raise the extracellular MC–LR concentration during the blooms. However, when cell lysis occurs at the end of the bloom, a large amount of accumulated MC–LR will be released into the water, suddenly and significantly increasing the concentration of MC–LR in the water.

### 3.3. Relationship between Photosynthetic Capacity and MC–LR Production of M. aeruginosa under Iron Variation

**Figure 4:**
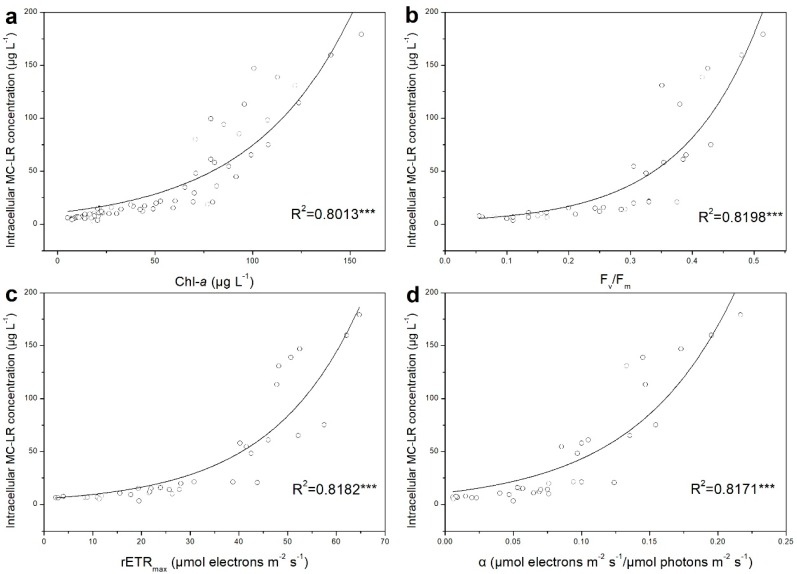
Relationship between physiological activity and intracellular MC–LR concentration of *M. aeruginosa* FACHB–905 ((**a**): Chl-*a* (chlorophyll-*a*) content, *n* = 70; (**b**): *F_v_/F_m_*, maximal quantum yield, *n* = 35; (**c**): *rETR_max_*, maximal relative electron transport rate, *n* = 35; (**d**): *α*, photosynthetic efficiency, *n* = 35). Both the R^2^ and significance level are indicated; *** *p* < 0.001.

**Figure 5:**
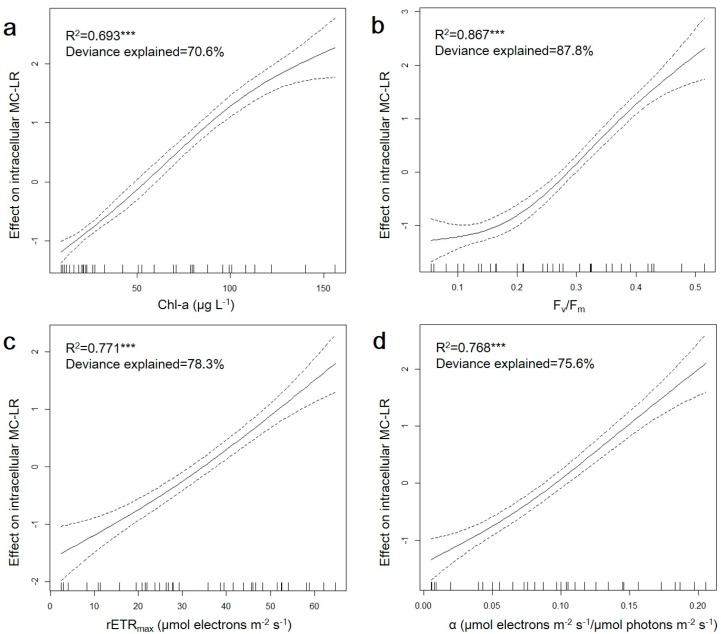
Chl-*a* (chlorophyll-*a*) (**a**), *F_v_/F_m_* (**b**), *rETR_max_* (**c**) and *α* (**d**) effect curves from a GAM model fitted to intracellular MC–LR concentration data (solid lines), with two 95% confidence bands (dashed lines). R^2^, significance level and explained deviation are indicated; *** *p* < 0.001.

According to previous studies, the MC concentration was found to increase linearly under light limiting conditions, while decrease under saturating light intensities [74]. It is also reported that the majority of intracellular MC is linked to the thylakoid region where photosynthesis takes place [75,76], indicating that MC production might potentially correlate with cyanobacterial photosynthesis. Interestingly, in our study, a significant exponential relationship between photosynthetic parameters and intracellular MC–LR concentration was observed (Figure 4) under iron variation. As an important participant in photosynthetic process, the Chl-*a* content was significantly correlated with intracellular MC–LR concentration (R^2^ = 0.8013, *n* = 70, *p* < 0.001), which was also observed in previous studies [77,78]. In terms of photosynthetic capacity, *F_v_*/*F_m_*, *rETR_max_* and *α* all showed significant positive correlations with the intracellular MC–LR concentration (R^2^ = 0.8198, n = 35, *p* < 0.001; R^2^ = 0.8182, *n* = 35, *p* < 0.001; R^2^ = 0.8171, *n* = 35, *p* < 0.001, respectively). Furthermore, in order to quantitatively reveal the relationship between photosynthetic capacity and MC production, a GAM model was applied (Figure 5). The degrees of freedom were 2.42, 2.67, 1.50 and 1.80 for Chl-*a*, *F_v_/F_m_*, *rETR_max_* and *α*, respectively, suggesting that there was a non–linear relationship between photosynthesis–related parameters and intracellular MC–LR concentration. The degrees of freedom explain contributions of photosynthetic parameters to MC production response and the larger the degree of freedom, the more important the parameter [51]. The statistical results demonstrated that all three photosynthetic capacity parameters were non–linearly and significantly (*p* < 0.001) related to intracellular MC–LR variation and explained more variation than the Chl-*a* content, statistically implying that the photosynthetic capacity can be applied to predict MC production in toxic cyanobacteria. Our results are in good agreement with previous studies which reported that MC production was positively correlated with photosynthetic capacity in toxic *M. aeruginosa*, when light was lower than saturating intensity [74,79]. As the light saturation intensity for *M. aeruginosa* is approximately 32 μmol photons m^−2^ s^−1^ [80], the light setup in our experiment (30 μmol photons m^−2^ s^−1^) was considered as non–saturating.

At the cellular level, *F_v_/F_m_* and *rETR_max_* exhibited greater links with MC production in our study. Being the vital parameters of energy transfer and electron transportation in cells, the variation of *F_v_/F_m_* and *rETR_max_* implied an alteration of electron transport chain, which may lead to oxidative stress and accumulation of superoxide radicals in cells [81]. The effective quantum yield in PS II (*ϕPSII*) is calculated with the formula (*F_m_ − F_0_*)/*F_m_*, where F_0_ is linked to PS II reaction center closure [66]. The relative electron transport rate (*rETR*) can be derived by the product of *ϕPSII*, and its rapid light curve (RLC) was composed of a linear rise phase and a plateau phase, the relative value of which is *rETR_max_*. In our study, the accumulated electron resulted from iron variation affected PS II reaction center closure, leading to the increase of *ϕPSII* and eventually augmenting *rETR* and *rETR_max_*. The increase of *F_v_*/*F_m_* and *rETR_max_* suggested that more electron flux passed via the electron transport chain [56]. Then, the primary electron acceptor was subsequently reduced since prior to its re–oxidization, the primary acceptor lost its active function as an effective transporter when receiving an electron. Under this circumstance, excessive excitation energy at PS II was dissipated via a charge–recombination reaction, and such non–assimilatory dissipation of excitation can generate singlet oxygen that would lead to photo damage of PS II reaction centers [82]. Moreover, the formation of superoxide radicals was also reported to co–occur with iron variation due to its influence on iron–mediated Fenton reactions, resulting in the disorder of redox status and damage to cellular metabolism and structure [24]. Since a specific and covalent binding between MC and proteins was found to be strengthened and the sensitivity of MC–deficient mutants increased under oxidative stress [21,83], it can be deduced that the photosynthetic capacity, which may indicate redox status of cyanobacteria cells, is inherently correlated with MC production [44].

Besides, at the genetic level, MCs are synthesized by MC synthetase, which is encoded by the *mcy* gene cluster, and the expressions of which are often considered as an energy consumption process [41,84]. Interestingly, adenine triphosphate (ATP) production is one of the most important processes in cyanobacteria photosynthesis and is responsible for the energy production in cells [68]. Consequently, the change in photosynthetic capacity would influence the energy sensors which participate in regulation of gene expressions and eventually affect MC production in *M. aeruginosa* through the alteration of transcription of the *mcy* genes and the genes encoding MC–related proteins. According to previous studies, a decline in *mcy* expressions co–occurred with the absence of photosynthetic redox signaling from the electron transfer chain [85] and higher ATP synthase was observed in non–toxic strains compared with toxic strains [19], further indirectly confirming the close relationship between photosynthetic capacity (or ATP production) and MC production. In addition, it is also noted that the Nicotinamide Adenine Dinucleotide Phosphate (NADPH)–dependent reductases, which are important participants in energy transformation process, were differentially expressed in the MC–deficient mutant [21]. Furthermore, since the *mcy*H gene, which is intimately associated with MC biosynthesis pathway, was reported to be related to the binding site of ATP in cyanobacterial cells [43], the variation of ATP production would definitely alter *mcy*H expression and further affect MC production in *M. aeruginosa*, demonstrating that MC production greatly relies on ATP production in photosynthesis of *M. aeruginosa*.

It is commonly recognized that photosynthesis and cyanobacterial growth are controlled by nutrients, which were often reported to be associated with MC production. Therefore, our study proposes the hypothesis that iron variation indirectly influenced MC production through its direct alteration of redox status and energy production in photosynthesis process. As a result, in the future, more attention should be paid to the involvement of cyanobacterial photosynthesis in the regulation of MC production by iron, especially through proteomic analysis.

## 4. Conclusions

Our results demonstrated that iron addition can promote cell density and protein content, as well as the Chl-*a* content in *M. aeruginosa*, and the three photosynthetic parameters (*F_v_*/*F_m_*, *rETR_max_* and *α*) were also up–regulated in iron–containing exposures, peaking under the 60 μM exposure. Moreover, the favorable iron concentration for intracellular MC–LR production was also 60 μM rather than higher concentrations, and few cells were ruptured under iron variation according to the invariant extracellular MC–LR in the medium. Thus, it can be concluded that iron addition can stimulate growth and metabolic characteristics in *M. aeruginosa*, including photosynthetic capacity and MC–LR production, but not in a dose–dependent manner. In addition, a significant positive correlation between photosynthetic capacity and MC production was observed, and *F_v_/F_m_* and *rETR_max_* were the most important parameters contributing to the variation in the intracellular MC–LR concentration. Since the electron transport chain (*F_v_/F_m_* and *rETR_max_*) was affected by iron variation, ATP production would be inhibited and *mcy* gene expression, which is an energy consumption process, would be subsequently altered, indicating that MC production greatly relies on redox status and energy metabolism of photosynthesis in *M. aeruginosa*. In consequence, more attention should be paid to the involvement of photosynthesis of *M. aeruginosa* in the regulation of MC production by iron variation in the future.
